# Integrative scRNA-seq and transcriptomic analysis initially reveals monocyte/macrophage activation drives EV-A71-induced immune dysregulation and neural injury in severe HFMD

**DOI:** 10.3389/fimmu.2025.1620633

**Published:** 2025-08-21

**Authors:** Muqi Wang, Meng Zhang, Huiling Deng, Yufeng Zhang, Chenrui Liu, Yuan Chen, Chuting Zhang, Wen Zhang, Xiaoli Jia, Shuangsuo Dang, Yaping Li

**Affiliations:** ^1^ Department of Infectious Diseases, Xi’an Jiaotong University Second Affiliated Hospital, Xi’an, China; ^2^ Department of Infectious Diseases, Xi ‘an Children’s Hospital, Xi’an, China; ^3^ Department of Laboratory Medicine, Xi’an Jiaotong University Second Affiliated Hospital, Xi’an, China

**Keywords:** EV-A71, innate immunity, monocyte/macrophages, severe HFMD, nerve damage

## Abstract

**Objective:**

Enterovirus 71 (EV-A71) is a major pathogen of severe hand, foot and mouth disease (HFMD) in children, but the mechanism by which it develops into severe HFMD remains unclear, especially the role of macrophage-mediated immune dysregulation.

**Methods:**

Bioinformatics tools were utilized to analyze the transcriptome sequencing results of peripheral blood monocytes (PBMCs) infected with different titers of EV-A71 at various time points. Single-cell sequencing technology was used to sequence obtained PBMCs from a severe HFMD patient due to EV-A71 and a healthy control. Macrophages infected with EV-A71 were collected for transcriptomic analysis, and were indirectly co-cultured with nerve cells to observe their inhibitory effects on nerve cells.

**Results:**

Single-cell RNA sequencing (scRNA-seq) revealed that EV-A71 infected severe HFMD patient had higher monocyte and macrophage ratio (18.50% vs. 8.85%), especially classical (64.59% vs. 57.24%) and non-classical (32.23% vs. 23.90%) monocytes, and a lower pDC (1.19% vs. 12.01%) and monoDC (1.98% vs. 6.80%) in EV-A71 infected severe HFMD patient. Dynamic analysis of PBMCs infected with EV-A71 isolates (mild, moderate and severe) and cell trajectory analysis indicated during infection, monocyte/macrophages were initially activated, followed by three groups of T cells and NK and B cells, M1 macrophage. High concentration of EV-A71 infected macrophage supernatant inhibited SH-SY5Y cell proliferation. ENSG00000285779, TICAM2, RPL13AP26 and HNF4G are significantly different in EV-A71 or inactivated EV-A71 infected macrophages than in control. ENSG00000264324, ENSG00000260643, ISLR2, CCR7, TENM4, INO80B-WBP1, BLOC1S5-TXNDC5 are potential genes about direct virus damage or viral RNA recognition in macrophages. GO annotation and KEGG analysis indicate that EV-A71 infection cause the changes of neural receptor-ligand binding pathway, activation of specific immunity, calcium signaling pathway, and cell aggregation.

**Conclusions:**

Macrophages are activated early during EV-A71 infection, thus initiating specific immunity, which is closely related to the severe HFMD. The nerve damage pathway and calcium signaling pathway caused by EV-A71 virus infection of macrophages deserve to more attention.

## 1 Introduction

Enterovirus 71 (EV-A71) is one of the main pathogens causing hand, foot and mouth disease (HFMD), which has a neurotropic nature and tends to induce central nervous system damage. EV-A71 has replaced poliovirus as the most important central neurotropic pathogen in the world (1). Severe HFMD manifests with significant central nervous system impairments, which include a spectrum of complications. Critical HFMD progresses rapidly, with a notably low treatment success rate. Research by Chinese scholars has revealed that the median duration from onset of HFMD to fatality in children is approximately 3.5 days, with the time from diagnosis to death decreasing alarmingly to only 1.5 days (2). Patients with severe disease have potential long-term neurodevelopmental effects even when they enter the recovery phase (3). The severity of HFMD, along with its complications and severe long-term effects, significantly contributes to the disease’s overall burden in terms of quality-adjusted life years (4, 5). Thus, it is necessary to explore in depth the pathogenesis of severe HFMD for early diagnosis and treatment.

Current research indicates that cellular immunity, humoral immunity, and cytokines are involved in the progression and tissue damage that occur in most viral infectious diseases (6). Studies also show that the main pathogen leading to severe and critical HFMD is EV-A71, with no changes in expression of enterovirus genes. Therefore, the incidence, progression, and prognosis of HFMD are closely related to the state of immune function (6). EV-A71 infection can lead to cellular immune dysfunction and massive cytokine production, suggesting extremely complex immune responses in the body (7). However, the specific classifications and subgroups of immune cells involved, as well as the dynamic changes in immune cells during the disease process, have not yet been determined. Single-cell sequencing technologies that can delineate immune-cell profiles that classify immune cells under a variety of conditions will be critical for understanding the pathogenesis of infectious diseases (8, 9). In particular, severe infections caused by EV-A71 can lead to profound neurologic damage, likely because the virus is able to evade the innate immune response and infiltrate the central nervous system (10). Numerous studies have shown that the immune response to EV-A71 infection is complex and unclear (11–13).

In the progression of inflammatory response induced by viral infection, macrophages recognize various components of pathogens through mechanisms such as pattern recognition receptors (PRRs), can initiate inflammatory response, phagocytic pathogens, and initiate specific immunity through antigen presentation, thus affecting the immune response after viral infection (14). To further explore the role of macrophages in the severity of EV-A71 infection, public data sets of peripheral blood monocytes (PBMCs) transcriptome sequencing with different severity of infection collected at different infection times and single-cell sequencing technology were used to study peripheral blood of patients with severe HFMD and healthy individuals with EV-A71. Finally, cell experiments were conducted to determine the transcriptomic changes of macrophages infected with EV-A71 virus and its inhibitory effect on nerve cells. We aimed to identify the role and mechanism of macrophages in EV-A71 infection.

## 2 Methods

### 2.1 Analysis of 80 RNA-seq data from EV-A71-infected PBMCs in GSE135964

We retrieved the GSE135964 dataset from Gene Expression Omnibus (GEO), which comprises 80 RNA-seq samples, to identify potential mediators that may account for severity-dependent differences in EV-A71 infection. This dataset includes RNA-seq data from mild, moderate, or severe EV-A71-infected PBMCs collected from four individuals at multiple time points post infection: 0, 6, 12 and 24 hours post infection (15). Infiltration of immune cells in 80 samples in the GSE135964 dataset was investigated using the TIMER 2.0 web server (http://timer.cistrome.org/), which is an online tool for exploring specific genes related to immune infiltrating cells (16). We analyzed the relative proportions of immune cells using various algorithms (TIMER, xCell, MCP-counter, CIBERSORT, EPIC and quanTIseq) in TIMER 2.0. Statistical significance was assessed through two-way ANOVA, with a P value threshold of less than 0.05.

### 2.2 Participants

This study was approved by the Medical Ethics Committee of Xi’an Jiaotong University Second Affiliated Hospital and Xi’an Children’s Hospital, Xi’an, China. Informed consent was obtained from parents of all participants. The diagnostic criteria for HFMD were established according to the clinical diagnostic criteria in Guidelines for Diagnosis and Treatment of HFMD (2018 Edition) (17) formulated by the Ministry of Health of the People’s Republic of China, with etiological confirmation established through a positive EV-A71 test from the child’s anal swab. Severe HFMD is characterized by the following: involvement of the nervous system (stage 2); symptoms such as diminished spirit, lethargy, and no progression to cardiopulmonary or cerebral failure. A female child with severe hand-foot-mouth disease caused by EV-A71 admitted to Xi ‘an Children’s Hospital was collected as a case, and a healthy child matched for gender and age as a control. Their peripheral blood mononuclear cells (PBMCs) were isolated for single-cell sequencing.

Both subjects had full-term natural births, were breastfed to 1 year of age, and did not receive EV-A71 vaccinations. The affected child presented with a peak temperature of 39°C, which had persisted for two days, with rashes developing post fever, accompanied by nervous system symptoms such as easy excitability and limb trembling. There were no observed symptoms related to the respiratory or circulatory system. Anal swab results for EV-A71 were positive. Peripheral blood sampling was conducted on the third day after illness onset, and the child was hospitalized for six days. Coinfection with Epstein–Barr virus, Mycoplasma, or Chlamydia were ruled out. Laboratory results indicated an elevated hypersensitive C-reactive protein (hs-CRP) level at 11.36 mg/L, an increased erythrocyte sedimentation rate (ESR) of 26 mm/h, normal procalcitonin (PCT) at 0.07 ng/mL, normal fasting blood glucose (FBG) at 3.88 mmol/L, creatine kinase (CK) at 82 U/L, and creatine kinase isoenzymes (CK-MB) at 24 U/L. The detailed clinical information and peripheral blood cell counts for both subjects are presented in **Table 1**.

**Table 1:** The basic clinical data and peripheral blood cell count of the two subjects.

Sample	Case	Control
Age(months)	23	18
Gender	female	female
Height(cm)	83	78
Weight(kg)	11	10.2
White blood cells(×10^9/L)	7.47	9.02
Neutrophil(×10^9/L)	1.1	1.84
Lymphocyte(×10^9/L)	5.53	6.41
Monocyte(×10^9/L)	0.6	0.48
Neutrophil percentage(%)	14.7	20.4
Lymphocyte percentage(%)	74.1	71.1
Monocyte percentage(%)	8.1	5.3
Red blood cells(×10^12/L)	4.09	4.9
Hemoglobin(g/L)	110	131
Platelet(×10^9/L)	299	270

### 2.3 PBMCs were extracted for single-cell sequencing

For single-cell sequencing, peripheral venous blood (2 ml) from the severe HFMD patient and the control was collected into heparin anticoagulant tubes. PBMCs were then isolated via Ficoll density gradient centrifugation (Lymphocyte Separation Medium, Applygen, Beijing). Cell count and viability were assessed using an automated cell counter to ensure that the single-cell suspension was well dispersed, had high cell viability, and had an accurate count. We immediately subjected freshly extracted PBMC to 10× single-cell sequencing by Biotechnology Co., Ltd. The entire process includes single-cell counting, single-cell GEM production, reverse transcription, cDNA amplification, library construction, library quality testing, and superior sequencing. Concentration required for library quality testing >10ng/ul, and the fragment length is concentrated between 350-500bp.

Cell Ranger software was used for initial quality control, including cell integrity assessment, alignment to the reference genome, and quantification of gene expression from the raw sequencing data. The basic situation and original data of the two samples obtained is shown in the **Supplementary Materials**. Seurat was utilized for further expression quality control, discarding genes expressed in fewer than three cells. Cells expressing more than 6,000 or fewer 2000 genes were excluded, as were those with mitochondrial gene-derived unique molecular identifiers (UMIs) constituting more than 20% of the total UMIs. We subsequently selected 2,000 genes with differential expression based on average expression and standardized variance across all cells. Cell similarity was computed using the PCA method; sample similarity was assessed through the t-distributed stochastic neighbor embedding (tSNE) and uniform manifold approximation and projection (UMAP) methods, with subsequent generation of cluster graphs. Marker genes were searched according to the difference in gene expression in each cluster, and we annotated the cell subpopulations according to human peripheral blood cell markers obtained from the CellMarker website (18) (http://xteam.xbio.top/CellMarker/). Following this, the distribution of these genes across clusters was assessed, and statistical analysis of gene expression within clusters was performed. Finally, enrichment of marker genes according to Gene Ontology (GO) analysis was analyzed using the cluster profiler tool. The expression statistics of the genes in the clusters were subsequently collated, and the marker genes were subjected to GO analysis using Cluster Profiler.

### 2.4 THP-1 cell-induced macrophage infection with EV-A71

The well-grown THP-1 cells were inoculated into a 6-well plate of RPMI-1640 medium containing 10% FBS and cultured in a 5% CO_2_ incubator at 37°C. Add the PMA dissolved with DMSO to the final concentration of 100 ng/mL and mix gently. The THP-1 cells were cultured in a 5% CO_2_ incubator at 37°C for 48 hours to induce differentiation into macrophage-like cells. After induction, the culture-medium was removed, cells were washed twice with preheated PBS, unadherent cells were removed, and fresh culture-medium was replaced.

EV-A71 (strain 87-2008 Xi’an Shaanxi, GenBank accession no.HM003207.1) was obtained from the Xi’an Centre for Disease Control and Prevention (Xi’an, Shaanxi, China). Inactivated EV-A71(VAG357) was obtained from KMD Bioscience (Tianjin, China). EV-A71 was thawed and diluted in medium to the desired number of infections (MOI 0.1). Infected cells: The culture medium induced by THP-1 was removed and diluted EV71 venom was added. Incubate in 37°C, 5% CO_2_ incubator for 1-2 hours, so that the virus is fully adsorbed. The venom was sucked out, the cells were gently washed with PBS twice to remove the unadsorbed virus, and the cell samples were collected for the next test when the culture was continued until 24h, and the supernatant was collected for further nerve cell co-culture. Macrophage and supernatant samples infected with inactivated EV-A71 and blank controls were collected using the same method.

Western Blot and real-time quantitative PCR were used to detect the infection effect. In Western Blot, the antibodies used were rabbit polyclonal antibody EV-A71 (KMD Bioscience Co., Ltd., China, PA2059, 1:500 dilution) and mouse monoclonal antibody β-actin (Affinity, T0022, 1: 20000 dilution), HRP labeled sheep anti-rabbit secondary antibody (Beyotime Biotechnology, China, A0208,1:1000), HRP labeled sheep anti-mouse secondary antibody (Proteintech, China, SA00001-1,1:10000), the membrane transfer conditions were 1.5A, 420s. In RT-PCR, the primer sequences of EV-A71 are forward:5’-GCAGCCCAAAAGAACTTCAC-3’and reverse:5’-ATTTCAGCAGCTTGGAG TGC-3’, with β-actin as the internal references. The primer sequences of β-actin are forward: 5’-AGCGAGCATCCCCCAAAGTT-3’ and reverse: 5’-GGGCACGAAGGCTCATCATT-3’. The primers were synthesized by the Shanghai Shenggong Biological Engineering Co., Ltd, and the real-time fluorescent quantitative PCR was performed according to the instructions of the SYBR^®^ Premix Ex Taq™ II (Takara, JPN) kit.

### 2.5 Indirect co-culture experiment of SH-SY5Y and THP-1 induced macrophages

SH-SY5Y cells at logarithmic growth stage and in good growth state were inoculated with 5×10^3^ cells/well into 96-well cell culture plates, cultured overnight in a 5% CO_2_ incubator at 37°C, and 200μl sterile PBS was added into the pores around the cell holes. The SH-SY5Y cells were divided into three experimental groups and one control group. The experimental groups included: SH-SY5Y cells cultured with macrophage supernatant containing live EV-A71; SH-SY5Y cells cultured with macrophage supernatant containing inactivated EV-A71; SH-SY5Y cells cultured with blank control macrophage supernatant (uninfected). The control group consisted of SH-SY5Y cells maintained in complete culture medium. For all experimental groups, supernatant concentration gradients were established at 10%, 20%, 30%, 40%, and 50%. Following overnight cell plating, the medium was replaced with SH-SY5Y-specific culture medium containing cryopreservation medium at the corresponding concentrations. Cells were further incubated for 24 and 36 hours. Subsequently, 10 μL of CCK-8 reagent was added to each well, followed by incubation at 37°C for 2 hours. Absorbance values (OD450) were measured using a microplate reader at a wavelength of 450 nm to assess cell viability.

### 2.6 RNA sequencing of macrophages infected with EV-A71

Total RNA was extracted using TRIzol, on-column DNase digestion to eliminate genomic DNA contamination. The extracted RNA samples were sent to Jingneng Biotechnology (Shanghai, China) for subsequent transcriptome sequencing. RNA purity and concentration were quantified via NanoDrop spectrophotometry (A260/A280 = 1.8-2.1; A260/A230 ≥ 2.0) and Qubit^®^ fluorometry (≥500 ng/μL), and RNA gel electrophoresis 28S:18S ≥ 1.5. For library preparation, RNA fragmentation (200-500 nt) and cDNA library size (300-500 bp inserts) were confirmed via Bioanalyzer, with library concentrations measured by Qubit^®^ dsDNA HS Assay. Post-sequencing metrics required ≥20 million raw reads per sample (Illumina platforms), Q30 scores ≥80%, and rRNA reads ≤5% (mRNA-seq).

Cleaned reads were aligned to a reference genome using STAR or HISAT2, requiring ≥85% alignment rate. Gene-level counts were generated with featureCounts or HTSeq, and normalized to transcripts per million (TPM) or fragments per kilobase per million (FPKM). Differentially expressed genes (DEGs) were identified using DESeq2 or edgeR, with significance thresholds set at |log2(fold change)| ≥1 and false discovery rate (FDR) ≤0.05. DEGs were annotated for GO terms and Kyoto Encyclopedia of Genes and Genomes (KEGG) pathways using ClusterProfiler, with enrichment significance defined as FDR ≤0.05.

### 2.7 Statistics

Statistical analyses were performed with SPSS 22.0 software (SPSS, Inc., Chicago, IL), R3.6.1 and figures were generated using GraphPad Prism 9 (GraphPad, United States). Continuous variables with a normal distribution are expressed as the mean ± standard deviation, and a t test was used for comparison of means. Variables with skewed distributions are expressed as medians (25%, 75%), and comparisons of means were performed with the Mann–Whitney U test. Count variables are expressed as counts (%), and the chi-square test was used to compare rates. The Pearson correlation coefficient test was applied for correlation analysis of two continuous variables with a normal distribution, and the Spearman rank correlation test was used for other variables. P < 0.05 indicated statistical significance.

## 3 Results

### 3.1 Immune cells that undergo significant changes after EV-A71 infection

**Figure 1:**
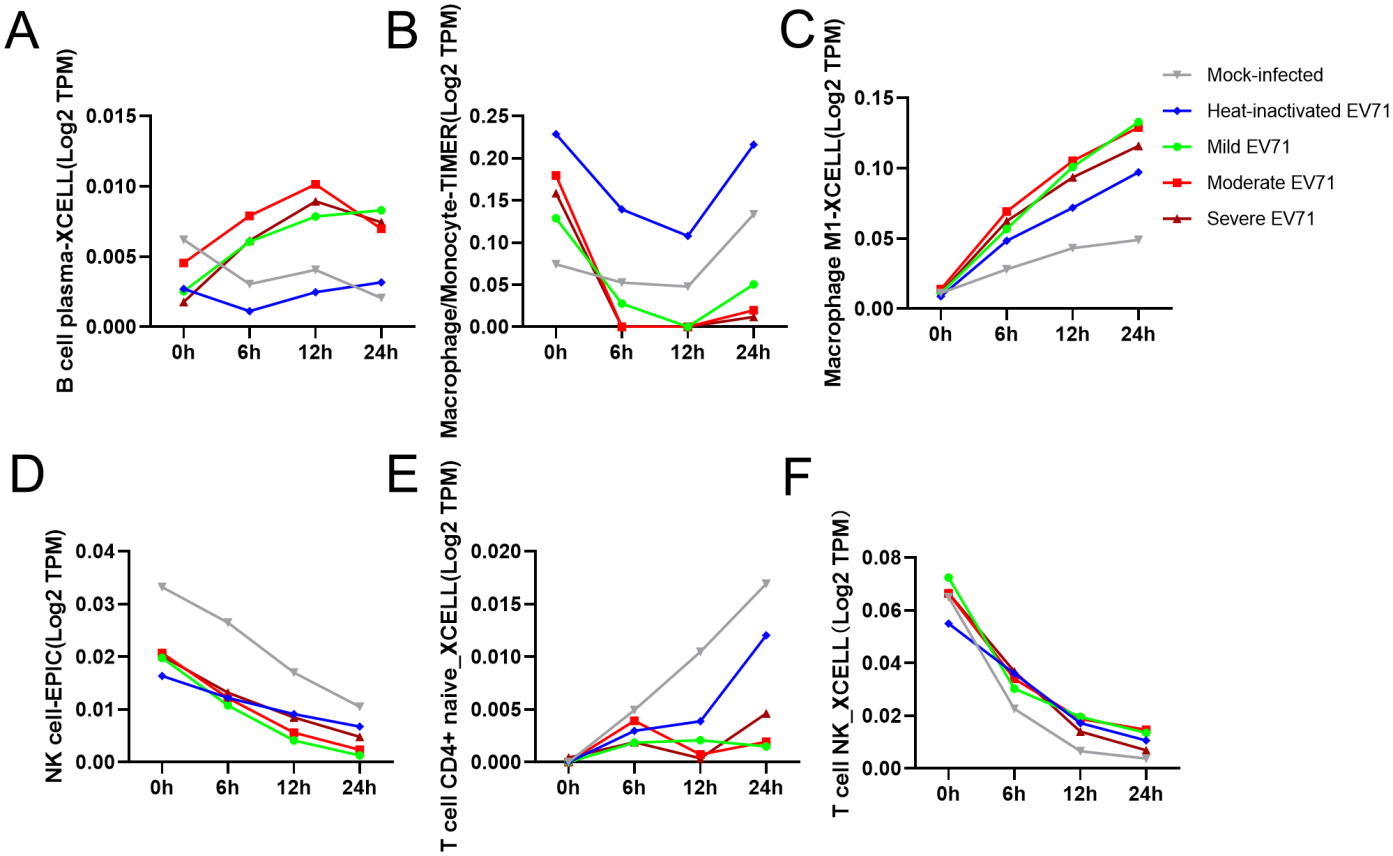
Distribution of immune cells according to the degree of EV-A71 infection and duration and correlation analysis of immune cell infiltration levels. **(A–F)** Numbers of immune cells with significant differences at different titers and infection times after simulated EV71 infection. TIMER, Tumor Immune Estimation Resource.

At the same time, we used TIMER 2.0 web server to study the immune cell infiltration of 80 samples in GSE135964 dataset. In the GSE135964 dataset, PBMCs from four donors were selected for analysis. These PBMCs were exposed to three EV-A71 viral strains, which represented mild, moderate, or severe infection, and samples were collected at 0, 6, 12 and 24 hours post infection. Mock-infected and inactivated EV-A71 isolates served as negative controls. RNA-seq data for a total of 80 samples were organized into 20 groups with 4 samples per group. Using the TIMER2.0 algorithm to predict the immune cell infiltration of PBMCs, a total of 119 variable indicators were obtained and a two-way ANOVA analysis was conducted. Among them, there were 7 significant related indicators (corrected P < 0.05) related to the severity of EV-A71 infection, 56 significant indicators related to the duration of EV-A71 infection (corrected P < 0.05), and a total of 7 indicators related to both the duration and severity of EV-A71 infection were listed in the **Table 2**. **Figure 1** revealed the presence of monocyte/macrophages and M1 macrophages following EV71 infection.

**Table 2:** The immune cells that showed significant changes in response to the severity and duration of EV-A71 infection were obtained by analyzing the dataset GSE135964.

Cell Types	F (Infection)	P (Infection)	F (Time)	P(Time)
Macrophage/Monocyte_TIMER	6.4838	0.00020978	3.4243	0.022702
Macrophage M1_XCELL	5.493	0.00077769	14.129	4.48E-07
NK cell _EPIC	4.9234	0.0016859	6.6543	0.000592
B-cell plasma_XCELL	4.1888	0.0046688	5.4335	0.002258
T-cell CD4+ naive_XCELL	3.2300	0.018177	18.472	1.32E-08
T-cell NK_XCELL	2.6217	0.043526	140.35	4.44E-27
NK cell_QUANTISEQ	2.6031	0.044706	5.5555	0.001971

The immune cell infiltration level of PBMC was predicted using the TIMER2.0 algorithm (TIMER, XCell, MCPCOUNTER, CIBERSORT, CIBERSORT-ABS, EPIC and QUANTISEQ). Two-factor variance analysis were used to investigate the effects of infection duration and severity on the levels of immune cells. F refers to the variance value, P refers to the significance test value, and in the table, it represents the variance value and P value corresponding to the influence of one independent variable (severity or time) on the immune cell level when the other independent variable is fixed.

### 3.2 Single-cell sequencing in the acute phase of severe EV-A71-related HFMD and healthy control

**Figure 2:**
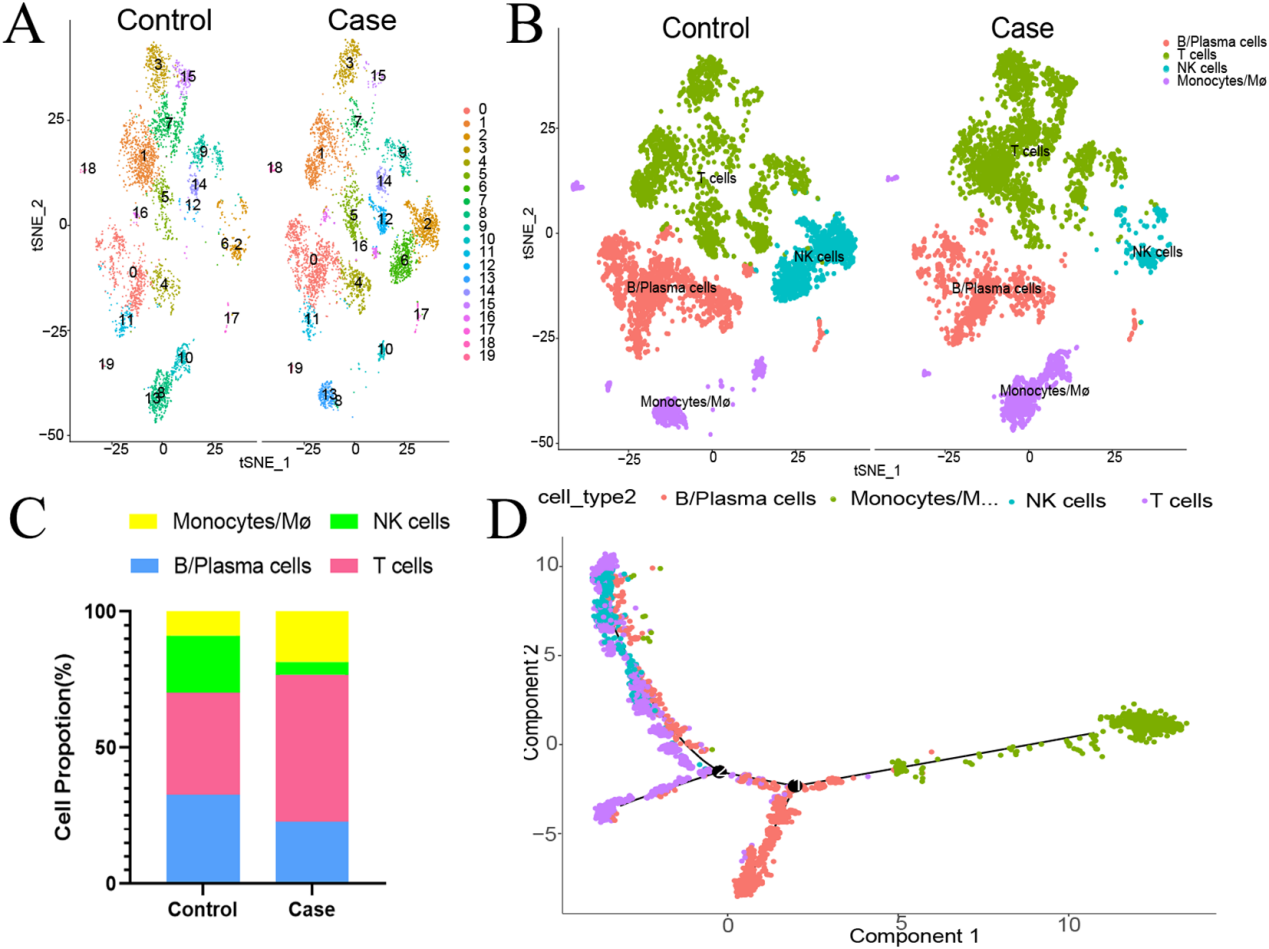
The proportion of peripheral blood mononuclear cells in the HFMD patient at the acute stage compared with that in the healthy control. **(A)** The distribution of PBMC subsets is shown. **(B)** Different types of immune cells are annotated. **(C)** Proportions of different types of immune cells. **(D)** Cell trajectory analysis suggested dynamic changes in and migration trajectories of immune cells during disease progression. NK cells, natural killer cells; Mø, macrophages.

**Figure 3:**
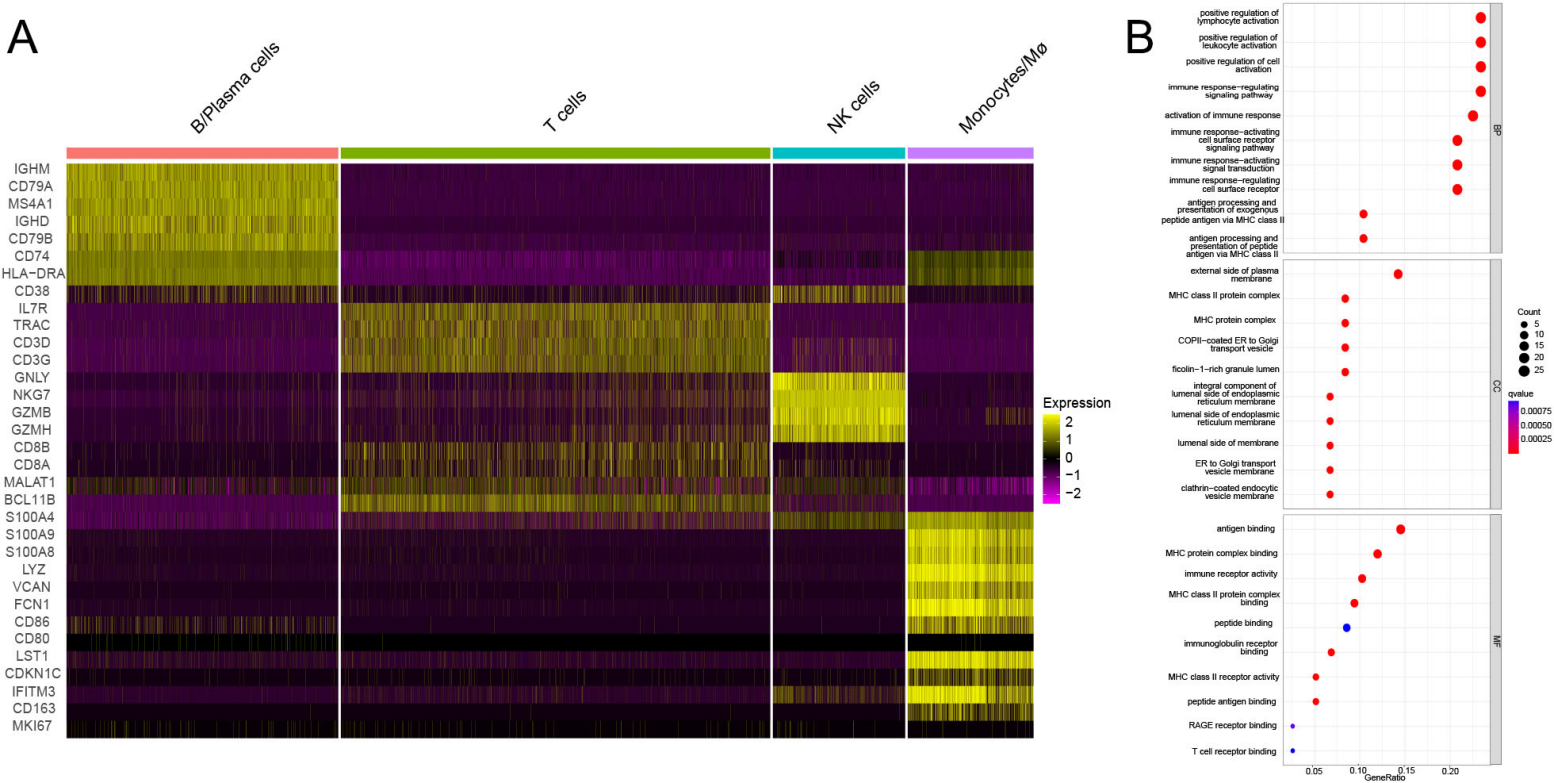
The DEGs of each cell subpopulation and their potential mechanism. **(A)** The marker genes of each cell subpopulation are shown. **(B)** GO analysis of DEGs with simulated temporal variation.

Single-cell sequencing of peripheral blood mononuclear cells was also conducted for the healthy control and for the patient in the acute phase of illness. The distribution of PBMC subsets is depicted in **Figure 2A**. Clusters 0, 4, 11, 16, and 17 were annotated as B/plasma cells due to MS4A1+CD79A+; clusters 1, 3, 5, 7, 9, 12, 14, and 15 were annotated as T cells due to CD3D+CD3E+; clusters 2 and 6 were annotated as NK cells due to NKG7+GNLY+; and clusters 8, 10, 13, 18, and 19 were annotated as monocyte/macrophages due to CD14+ (**Figure 2B**). The monocyte and macrophage ratio (18.50% vs. 8.85%) and T-cell ratio (54.04% vs. 37.43%) were greater in the patient group than in the control group, but the NK cell ratio (4.71% vs. 20.99%) and B-cell ratio (22.75% vs. 32.73%) were lower (**Figure 2C**). The dynamic changes and migration trajectories of immune cells during disease progression were tracked. Pseudosequential analysis and RNA velocity methods arrange each cell on the corresponding trajectory according to time-series gene expression and divide the sample into multiple differentiated cell groups according to gene expression status to generate an intuitive lineage development dendrogram, which can predict the differentiation and developmental trajectories of cells. Our findings indicate that monocyte/macrophages are the primary responders in the early stages of EV-A71 infection. These cells subsequently diverged into three branches, differentiating into T cells, NK cells and B cells, as illustrated in **Figure 2D**. The marker genes of each cell subpopulation are shown in **Figure 3A**. GO analysis of differentially expressed genes (DEGs) with simulated temporal variation revealed them to be mainly involved in lymphocyte activation, MHC complex formation and antigen binding (**Figure 3B**).

### 3.3 Further cluster annotation of monocyte/macrophages reveals greater proportions of classical monocytes and nonclassical monocytes in the EV-A71 patient than in the control

**Figure 4:**
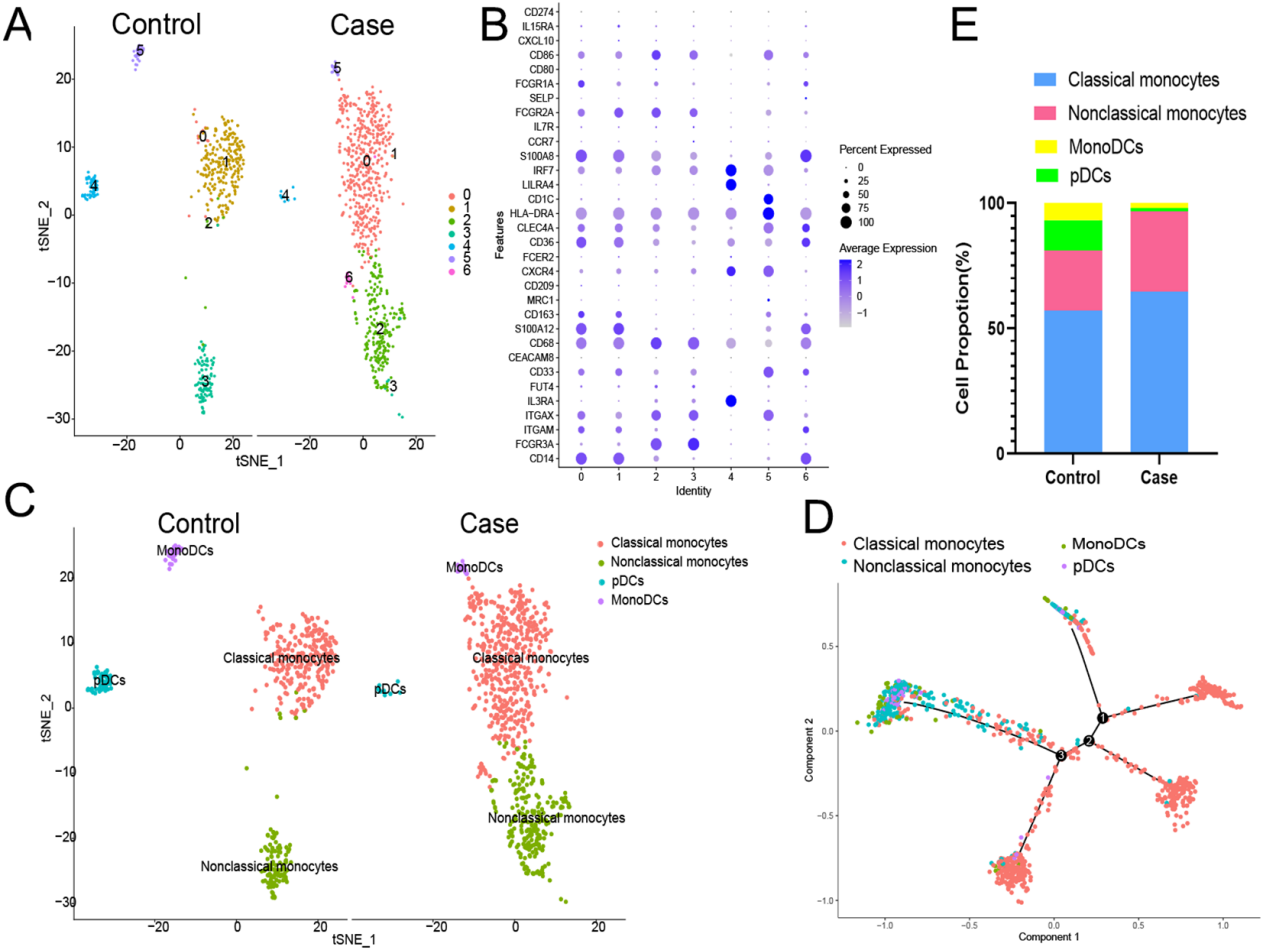
Further monocyte/macrophages cluster annotation. **(A)** Monocyte/macrophages were further classified into 7 subgroups. **(B)** Different types of monocyte/macrophages types are annotated. **(C)** Proportions of different types of monocyte/macrophages. **(D)** Cell trajectory analysis of monocyte/macrophages. pDCs, plasmacytoid dendritic cells; monoDCs, monocyte-derived dendritic cells.

**Figure 5:**
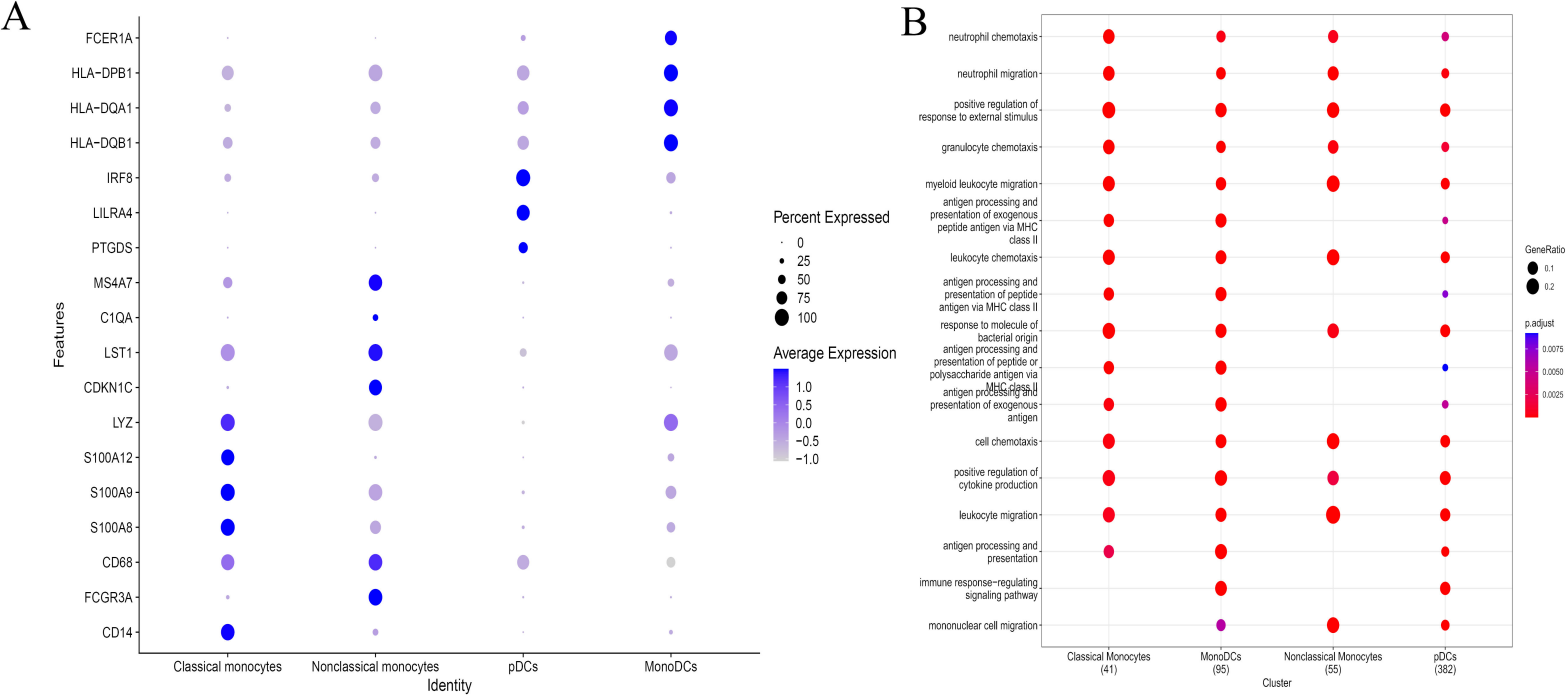
The DEGs of different types of monocyte/macrophages and their potential mechanism. **(A)** Expression of differentially expressed genes in different types of monocyte/macrophages. **(B)** GO analysis showing the functional annotation of monocyte/macrophages subset.

The above results suggested that monocyte/macrophages may play an important role in the process of EV71 infection, so we further explored to determine the specific type of monocyte/macrophages changed. Monocyte/macrophages were further classified into 7 subgroups (**Figure 4A**). Clusters 0, 1 and 6 were annotated as classical monocytes due to CD14+CD16-; clusters 2 and 3 were annotated as nonclassical monocytes due to CD14-CD16+; cluster 4 was determined to be plasmacytoid dendritic cells due to LILRA4+IRF7+; and cluster 5 was annotated as monocyte-derived dendritic cells due to CD1C+HLA-DRA+ (**Figure 4B**). Similarly, the classical monocyte ratio (64.59% vs. 57.24%) and nonclassical monocyte ratio (32.23% vs. 23.90%) were greater in the patient with EV-A71-related HFMD than in the healthy control, with lower pDC (1.19% vs. 12.01%) and monoDC (1.98% vs. 6.80%) ratios (**Figure 4C**). Cell trajectory analysis suggested that classical monocytes and monoDCs were the starting points and subsequently led to branching of nonclassical monocytes and pDCs (**Figure 4D**). The classical monocytes mainly expressed S100A8, S100A9, S100A12, and LYZ, whereas the nonclassical monocytes highly expressed CDKN1C, LST1, C1QA and MS4A7. Plasmacytoid dendritic cells (pDCs) mainly expressed PTGDS, LILRA4 and IRF8; mononuclear dendritic cells (monoDCs) highly expressed HLA-DQA1, HLA-DQB1, HLA-DPB1 and FCER1A (**Figure 5A**). GO analysis was performed on the DEGs in each cell subgroup to obtain functional annotations for each subgroup, as shown in **Figure 5B**.

At the same time, we also analyzed the specific subtype changes of other types of T cells, B cells and NK cells, and obtained a large number of results (**Supplementary File S1**), which need to be further verified.

### 3.4 High concentration of EV-A71-infected macrophage supernatant inhibited SH-SY5Y cell proliferation

**Figure 6:**
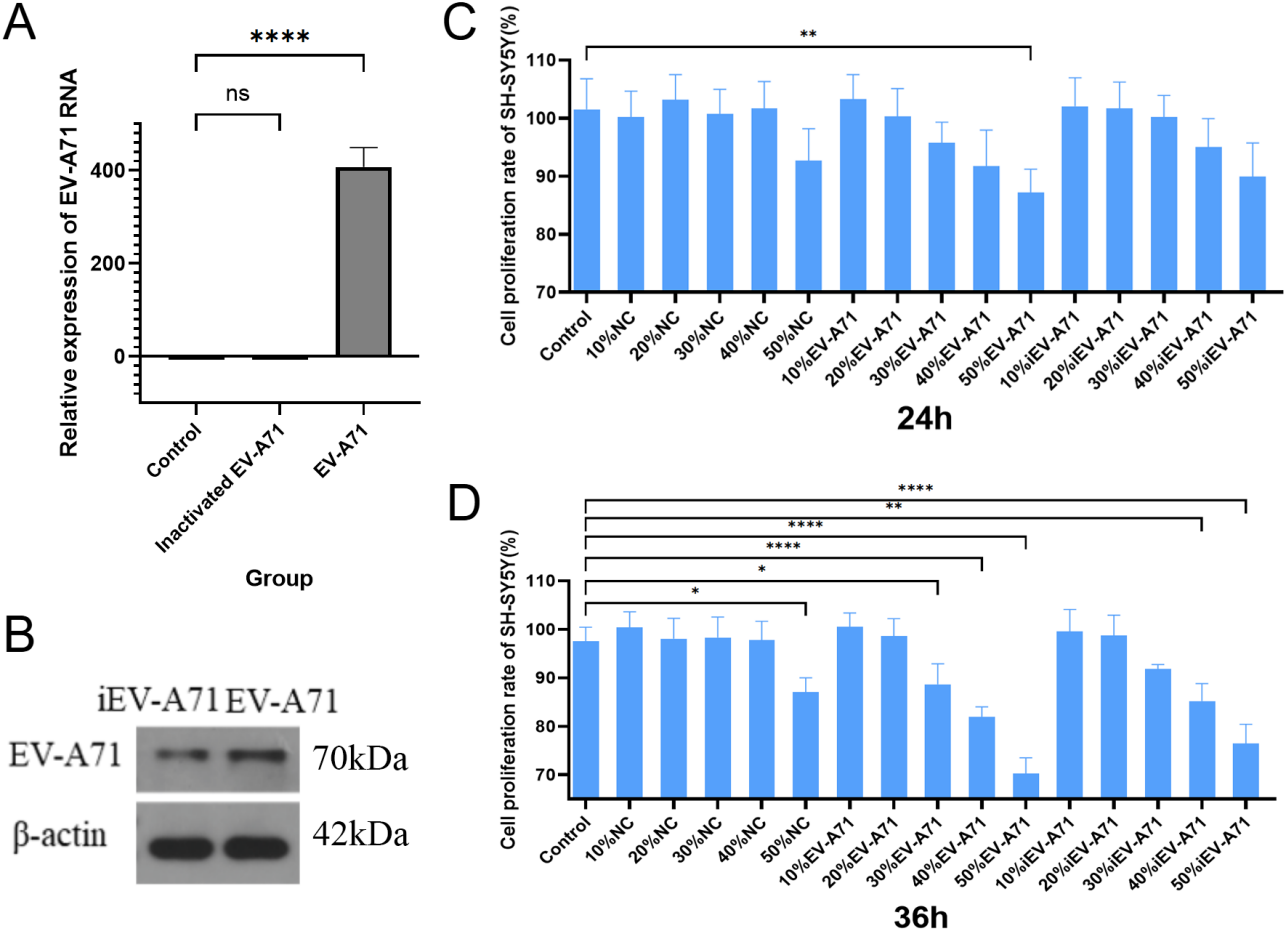
High concentration of EV-A71-infected macrophage supernatant inhibited SH-SY5Y cell proliferation. **(A)** EV-A71 RNA detection. **(B)** EV-A 71 protein detection. **(C)** The cell proliferation rate of the supernatant of EV-A71-infected macrophages co-cultured with SH-SY5Y for 24 hours. **(D)** The cell proliferation rate of the supernatant of EV-A71-infected macrophages co-cultured with SH-SY5Y for 36 hours. NC, normal control, macrophage supernatant co-cultured with SH-SY5Y; iEV-A71, inactivated EV-A71, the supernatant of inactivated EV-A71-infected macrophages co-cultured with SH-SY5Y; EV-A71, the supernatant of EV-A71-infected macrophages co-cultured with SH-SY5Y. *P<0.05, **P<0.01, ****P<0.0001; ns, P>0.05.

To evaluate the potential downstream effects of macrophage activation during EV-A71 infection, we conducted co-culture experiments with neuronal cells. As shown in **Figure 6A**, a large amount of EV-A71 virus RNA could be detected when RNA was extracted from EV-A71 group cells, while no viral RNA was detected in inactivated EV-A71 group. EV-A71 protein was detected in both EV-A71 group and inactivated EV-A71 group (**Figure 6B**). The results of CCK8(**Figures 6C, D**) indicated that high concentration of macrophage supernatant inhibited cell proliferation, and the macrophage supernatant infected by EV-A71 was the most obvious, and the inhibition effect was more obvious with longer co-culture time.

### 3.5 Transcriptome sequencing of EV-A71 infected macrophages

**Figure 7:**
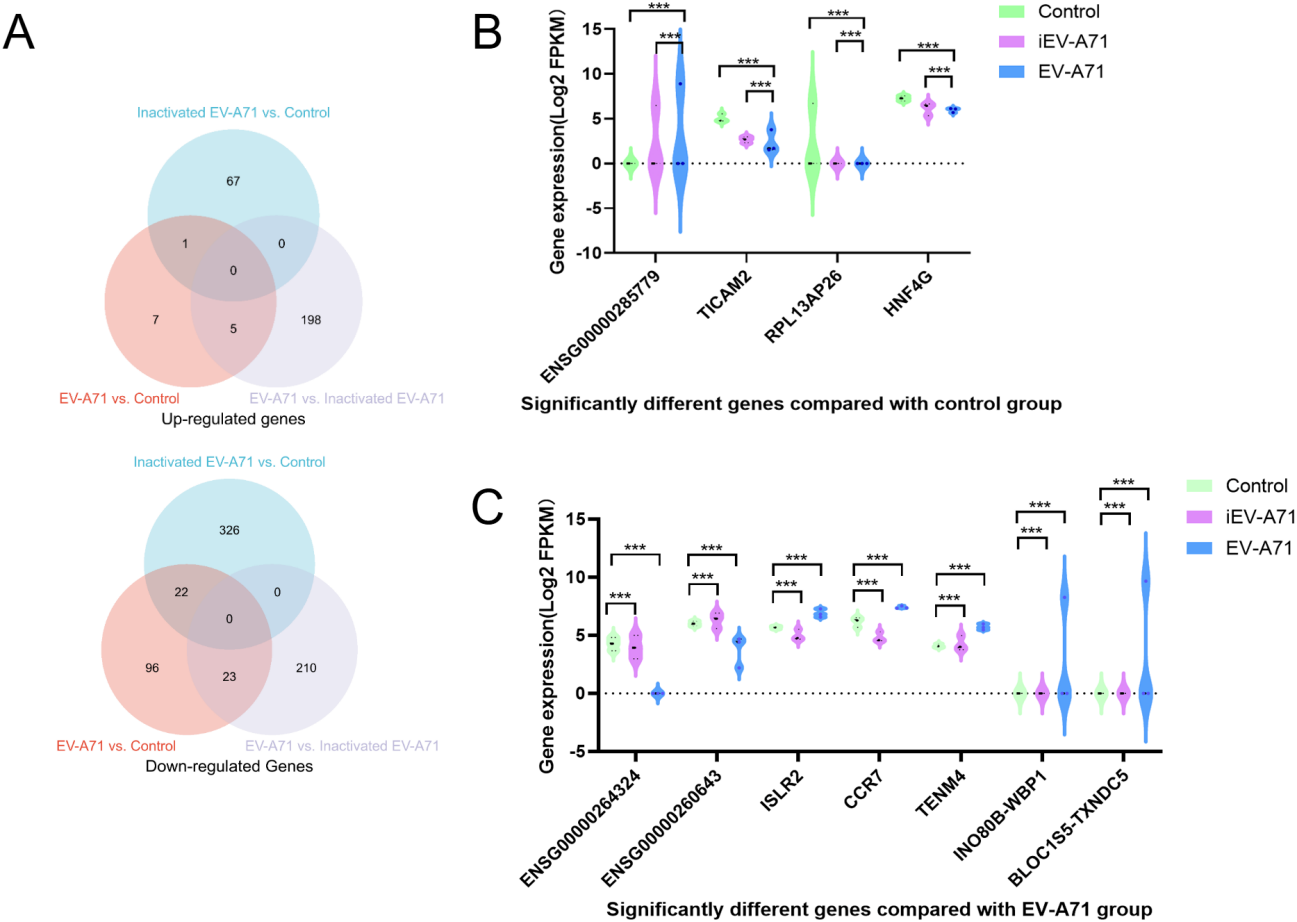
Transcriptome sequencing of EV-A71 infected macrophages. **(A)** The number of differentially expressed genes (DEGs) in each group. **(B)** Significantly different genes compared with control group. **(C)** Significantly different genes compared with EV-A71 group. ***P<0.001.

The RNA extracted from cells in all groups met the quality control requirements, and the mRNA expression of transcriptsets in all groups was compared. **Figure 7A** showed the number of differentially expressed genes in each group, and the genes significantly up-regulated/down-regulated by pair-to-pair comparison among the three groups, respectively (|log foldchange|>1, FDR<0.05).

Compared with the control group, ENSG00000285779 was up-regulated in both the infected EV-A71 group and the infected inactivated EV-A71 group, while TICAM2, RPL13AP26 and HNF4G were down-regulated in both groups. Considering that these genes were related to the innate immunity of macrophages caused by viral proteins, gene expression levels were shown in **Figure 7B**.

Compared with EV-A71 group, ENSG00000264324 and ENSG00000260643 were up-regulated in both normal group and infected inactivated EV-A71 group. Five genes were down-regulated in both groups (ISLR2, CCR7, TENM4, INO80B-WBP1, BLOC1S5-TXNDC5). These genes were considered to be related to direct virus damage or viral RNA recognition to initiate the innate immunity of macrophages, as shown in **Figure 7C**.

**Figure 8:**
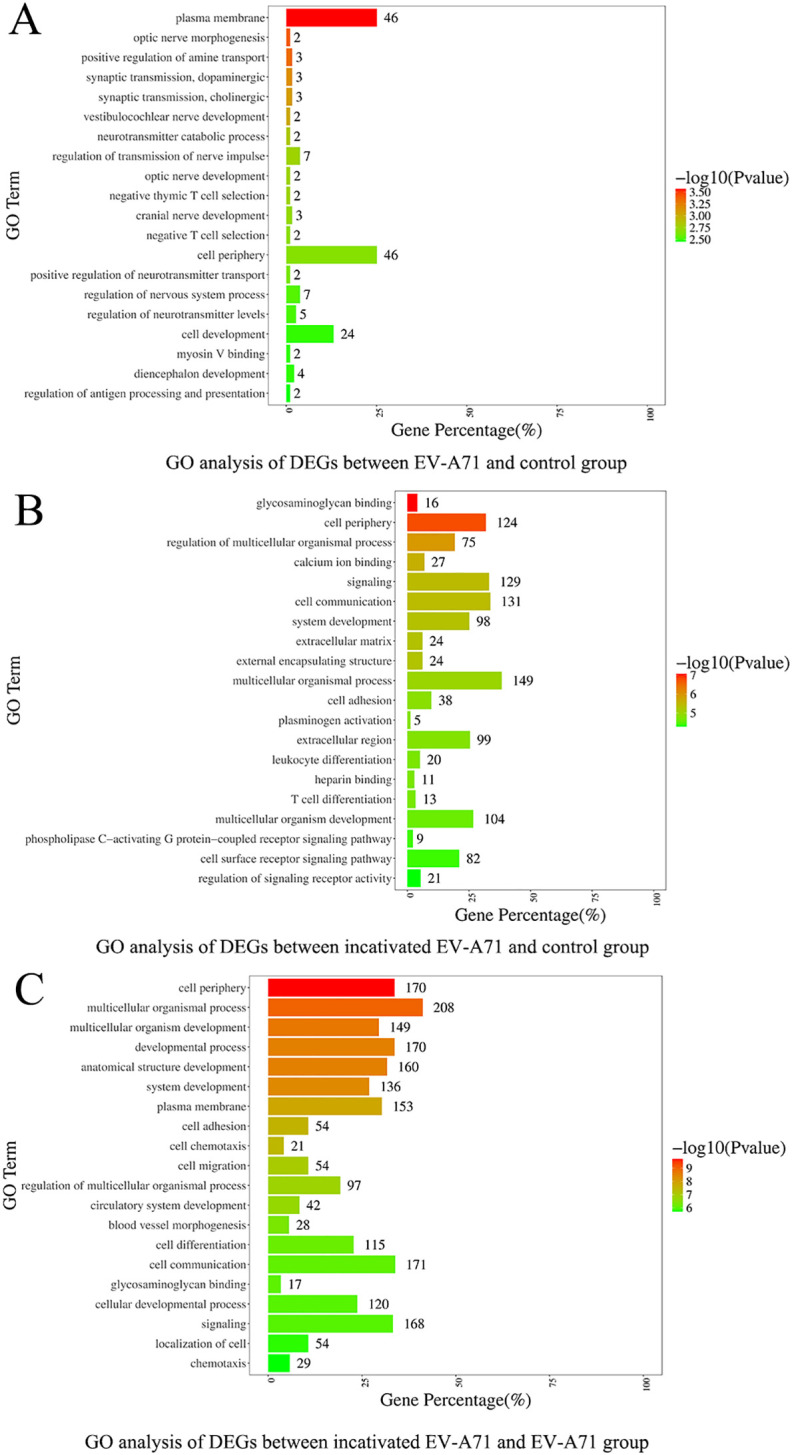
GO analysis of differentially expressed genes.

**Figure 9:**
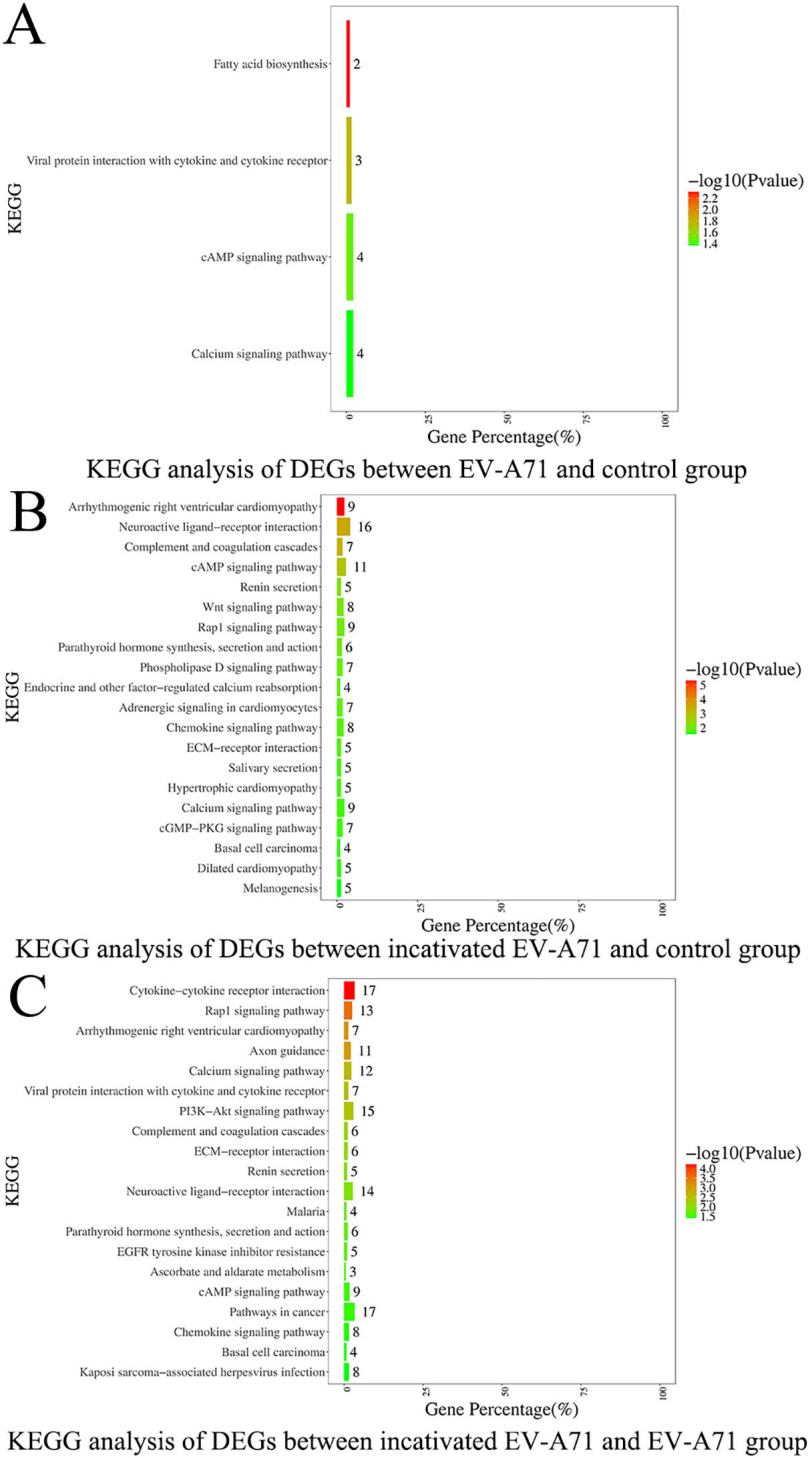
KEGG analysis of differentially expressed genes.

Three groups of macrophage transcriptional sequencing data of EV-A71, inactivated EV-A71 and blank control were compared in pairs. Genes (|log2(fold change)| >1 and P value <0.05) were analyzed for GO gene annotation, KEGG analysis, and protein interaction analysis. Significant changes in genes associated with cell periphery, regulation of antigen processing and presentation, and initiation of specific humoral and cellular immunity were observed after infection with EV-A71 and inactivated EV-A71 compared with normal controls in GO analysis (**Figure 8**). More notably, EV-A71 caused changes in the expression of genes associated with nervous system damage relative to normal controls, which were not seen in the inactivated virus group. KEGG analysis (**Figure 9**) found that compared with blank controls, after infecting macrophages with EV-A71 and inactivated EV-A71, calcium signaling pathway, viral protein interaction with cytokine and cytokine receptor, and cAMP signaling were activated pathway and other signaling pathways. Compared with inactivated EV-A71, EV-A71 significantly activated signal pathways such as Neuroactive ligand−receptor interaction and Cytokine−cytokine receptor interaction.

## 4 Discussion

This study reveals that monocytes/macrophages, especially M1-polarized cells, are early responders in EV-A71 infection and may play a dual role in antiviral defense and virus-induced neural damage. By integrating transcriptomic data and functional assays, we show that macrophage activation not only initiates innate immune responses but also contributes to neuronal injury, providing new insights into the immunopathogenesis of severe HFMD.

We firstly analyzed RNA transcriptome sequencing data of GSE135964, and found that monocyte/macrophages and M1 macrophages significantly varies with the duration of infection and the severity of the EV-A71 virus strain involved. Our single-cell RNA sequencing analysis showed a higher monocyte and macrophage ratio, especially classical and non-classical monocytes, and a lower pDC and monoDC in EV-A71 infected severe HFMD patient. The monocyte/macrophage system consists of three main cell types: monocytes, macrophages, and DCs. Monocytes are the precursors of macrophages and DCs; once monocytes leave the bloodstream and enter tissues, they mature into macrophages or DCs to perform their vital functions in the immune response. Indeed, these cells play a key role in maintaining tissue homeostasis during homeostasis and coordinating the onset and resolution of immune responses (19, 20). Classic mononuclear cells are thought to aid in phagocytosis, antibacterial activity and defense mechanisms in the host and play a role in blood vessel patrolling and surveillance (21). This indicated that the immune response was initiated after EV-A71 infection and that production of activated monocytes increased to exert antiviral effects. Monocyte/macrophages are sometimes not clearly distinguished. As shown in **Figure 5A**, classical monocytes and nonclassical monocytes also express the macrophage marker CD68 and the M1 macrophage marker CD86. Although both nonclassical monocytes and M1 macrophages are derived from monocytes in the blood, their functions and activation states are different. Nonclassical monocytes are usually associated with pathological conditions, whereas M1 macrophages are activated monocytes involved in specific immune responses that participate in antipathogen defense and inflammatory processes (22). The number of M1 macrophages, which are known to amplify inflammatory responses and facilitate pathogen clearance, is largely increased in the acute stage of infection, and with progression of virus infection-related diseases, macrophages polarize toward the M2 phenotype (7, 23). Our results indicate that EV-A71 infection stimulates increased monocyte production and even increases macrophage numbers and polarization toward the M1 phenotype, which are critical for establishing an effective antiviral response. This finding is in agreement with the findings of other studies on viral infections, which concluded that the susceptibility of monocytes to viruses changes upon differentiation into macrophages (24). These macrophages then undergo M1 polarization and exhibit heightened activation of innate immune and inflammatory pathways (25).

According to our single-cell sequencing results, monocyte/macrophages are likely activated first and subsequently trigger three branches, namely, B cells, NK cells and T cells. Moreover, GO functional annotation of each cell subgroup of these immune cells showed that their functions were consistent with their phenotypes. Through cell trajectory analysis, we found that classical monocytes and monoDCs act first in the infection process, followed by nonclassical monocytes and pDCs. This finding is consistent with the function and transformative relationship of the monocyte/macrophage system. These results indicate that there is not a single immune cell involved in EV-A71 infection but rather a collaborative response of multiple cells, and it is likely that the virus first activates some cells and, through these cells, activates other cells, thereby triggering a cascade of immune responses to clear the virus.

The specific mechanism of nerve injury caused by EV-A71 infection remains unclear, involving multiple aspects such as the virus breaking through the blood-brain barrier with neurotoxicity, immune response imbalance and pro-inflammatory factor storm, and mitochondrial damage (26, 27). The mechanism of macrophages in the process of immune response imbalance caused by EV-A71 infection and eventually nerve injury remains unclear. Previous studies showed that peripheral monocyte counts were lower in both absolute counts and frequencies in severe cases compared to mild cases (28). In order to clarify the damage to nerve cells caused by the immune response after macrophage infection with EV-A71, co-culture experiments using the neuronal SH-SY5Y cell line showed that the supernatant from EV-A71-infected macrophages markedly inhibited neuronal proliferation, indicating a cytotoxic or signaling-mediated effect likely contributing to the neurological manifestations seen in severe HFMD. Meanwhile, the inhibitory effect of the supernatant of macrophages that have been inactivated from EV-A71 infection on the proliferation of nerve cells indicates inactivated EV-A71 still contains intact viral proteins that may trigger innate immune recognition, though it lacks replication ability. To further explore the role of macrophages in EV-A71 pathogenesis, we conducted bulk transcriptomic analysis on EV-A71-infected macrophages. This revealed a distinct transcriptional signature characterized by the upregulation of genes such as TICAM2, CCR7, ISLR2, and TENM4, which are potentially involved in viral RNA recognition, neuroimmune communication, and axonal development, and the roles and mechanisms of TICAM2, CCR7, ISLR2, and TENM4 in EV-A71 infection or HFMD have not been reported. Notably, pathway enrichment analysis indicated significant alterations in calcium signaling and neuroactive ligand-receptor interactions, suggesting mechanisms by which infected macrophages may mediate neural dysfunction. These findings provide novel mechanistic insight into the interface between antiviral immune responses and virus-induced neural damage.

The limitation of this study lies in the single cell sequencing sample size of one female, and the biological heterogeneity between the public *in vitro* dataset and our *in vivo* case-control data, but the conclusion drawn from the results of comprehensive bioinformatics analysis, single-cell sequencing and macrophage cytological experiments is reliable. We also plan to systematically analyze macrophage-derived cytokines/chemokines using multiplex assays or RNA-seq in future work, which will provide mechanistic insights into their roles in EV71 pathogenesis.

In conclusion, the immune response triggered by EV-A71 infection is a complex response involving multiple cells, among which macrophages, especially M1 macrophages, are involved in the early immune response to EV-A71 infection and may be linked to subsequent neural injury. Furthermore, after EV-A71 not the inactivated EV-A71 infects macrophages, it will induce the activation of related genes such as neurotransmitter regulation and neural axon transmission. These findings are hypothesis-generating and provide a rationale for further mechanistic studies to elucidate the immune–neural interface in severe HFMD.

## Data Availability

Data are accessible at NCBI’s Gene Expression Omnibus (GEO) database (accession number GSE269965 and GSE304239).
