# Supplementary material for: Integrative scRNA-seq and transcriptomic analysis initially reveals monocyte/macrophage activation drives EV-A71-induced immune dysregulation and neural injury in severe HFMD
Source: Front Immunol. 2025 Aug 21;16:1620633. doi: 10.3389/fimmu.2025.1620633 (PMC12408647; doi:10.3389/fimmu.2025.1620633)
Supplement: Supplementary file 1 [file DataSheet1.docx]

**Further cluster annotation of T cells, B cells and NK cells**

**1.Further cluster annotation of B cells showed that plasmacyte ratio elevated in EV-A71 HFMD patients than in control**

B cells were further classified into 7 subgroups (Figure S1A). Cluster 0,1 and 3 were annotated as naïve B cells due to CD19+CD27-. Cluster 2 and 5 were annotated as memory B cells due to CD19+CD27+IgM+IgD+IgA+IgG+, and cluster 4 was determined to be cycling plasma cells due to MZB1+IGHG1+MKI67+TOP2A+, cluster 6 was annotated to be plasmacyte due to CD19-CD27+CD38+SDC1+ (Figure S1B-C). Naïve B cells mainly expressed TCL1A, CXCR4 and IL4R, while memory B cells highly expressed CRIP1, TNFRSF13B. Cycling plasma mainly expressed NKG7, GNLY, CCL5, GZMA and GZMB, while plasmacyte highly expressed IGHA1, IGHG1, JCHAIN and IGHG2 (Figure S1D). The levels of plasmacyte ratio (1.29% vs. 0.77%) and naïve B cells ratio (72.38% vs. 69.34%) in children with EV-A71 HFMD were higher than those in normal controls, while memory B cells (23.52% vs. 25.03%) and cycling plasma cells (2.80% vs. 4.86%) ratio slightly lower (Figure S1E). Cell trajectory analysis showed that plasma cells and circulating plasma cells played a role after naive B cells and memory B cells (Figure S1F). The differentially expressed gene GO analysis annotation of each B/Plasma cells subpopulation suggests the function of these cells as shown in Figure S1G.

**
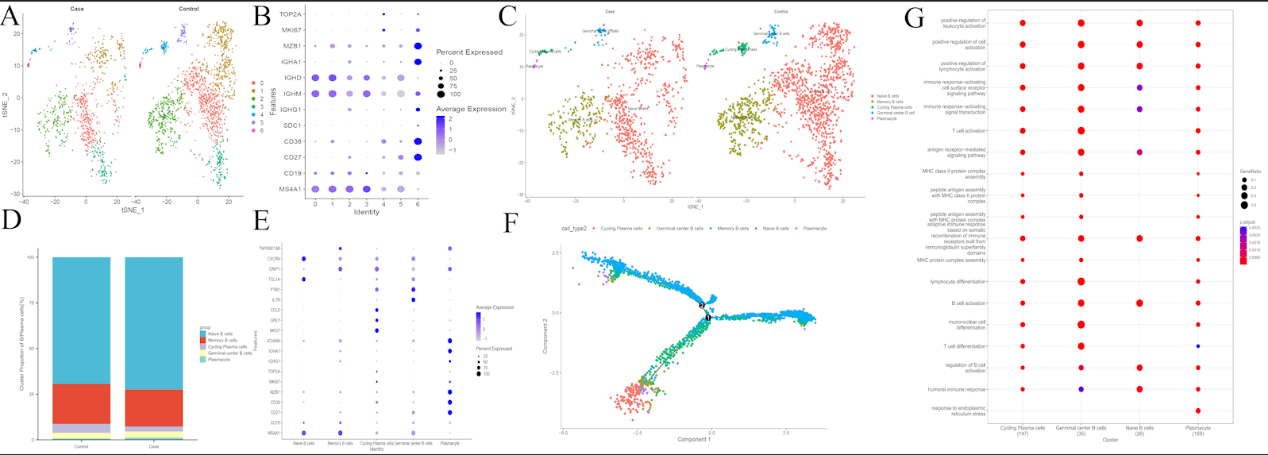
**

**Figure S1. Further cluster annotation of B cells**

**2. Further cluster annotation of T cells**

T cells were further classified into 9 subgroups (Figure S2A). Cluster 0 was annotated as CD4+ naïve T cells due to CD4+CD27+CCR7+LEF7+SELL+. Cluster 1 and 8 were annotated as CD8+ naïve T cells due to CD8A+CD8B+CD27+CCR7+ LEF7+SELL+, and cluster 2 was determined to be CD4+ memory T cells due to CD4+CD27-CD44+, cluster 3 was annotated to be CD4+ regulatory T cells due to CD4+FOXP3+IL2RA (CD25)+CTLA4+. Cluster 4 was determined to be NKT cells because of CD4-CD8-NKG7+GZMA+, and cluster 6 was annotated as γδT cells due to TRGV9+TRDV2+. Cluster 5 was annotated as CD8+ effector memory T cells due to CD8+IFNG+CD27loSELL(CD62L) loIL7Rlo and cluster 7 was annotated as CD8+ central memory T cells due to CD8+ CD27hiSELL(CD62L) hiIL7Rhi(Figure S2B-C). The levels of CD4+ memory T cells ratio (19.35% vs. 6.59%) and CD8+ central memory T cells ratio (7.77% vs. 1.81%) in children with EV-A71 HFMD were higher than those in normal controls, while CD8+ effector memory T cells (1.18% vs. 11.77%), CD4+ regulatory T cells (10.45% vs. 16.55%) and γδT cells (4.63% vs. 6.28%) ratio lower (Figure 4E). Cell ratios of naïve CD4+ T cells (31.56% vs. 30.39%), naïve CD8+ T cells (14.31% vs. 15.77%) and NKT cells (10.76% vs. 10.84%) are basically equal in patients and the control (Figure S2D). Cell trajectory analysis Marker genes of each cell subpopulation are shown in Figure S2E. Through cell trajectory analysis (Figure S2F), it can be concluded that the T cells mainly present in the early stage of EV-A71 infection are CD4+ Tn, CD4+ Treg and CD8+Tn, while CD4+ Tm, CD8+Tm, NKT and γδT cells in the late stage. The differentially expressed gene GO analysis annotation of each T cells subpopulation suggests the function of these cells as shown in Figure S2G.**
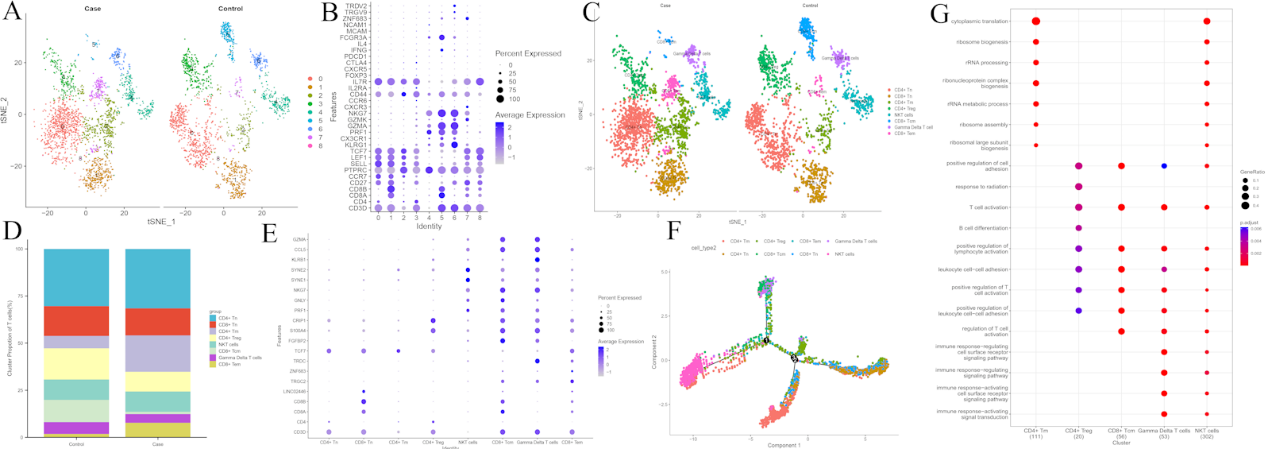
**

**Figure S2. Further cluster annotation of T cells**

**Tn: naïve T cells; Tm: memory T cells; Treg: regulary T cells; Tcm: central memory T cells; Tem: effectory memory T cells.**

**3. Further cluster annotation of NK cells**

NK cells were further classified into 4 subgroups (Figure S3A). Cluster 0,1 and 2 were annotated as CD56dimCD16+ NK cells due to NCM1(CD56)-FCGR3A(CD16)+. Cluster 3 and 5 were annotated as CD56brightCD16- NK cells due to NCM1(CD56)+FCGR3A(CD16)- (Figure S3B-C). The levels of CD56brightCD16- NK cells ratio (15.10% vs. 6.20%) in children with EV-A71 HFMD were higher than those in normal control (Figure S3D). CD56dimCD16+ NK cells mainly expressed FCGR3A, GZMB, NKG7, CD160 and PTPRC, while CD56brightCD16- NK cells mainly expressed NCAM1, KLRB1, IL2RB and IL7R (Figure S3E). Cell trace analysis suggests that CD56brightCD16- NK cells, in contrast to CD56brightCD16- NK cells may be a variant form of NK cells responding to infection (Figure S3F). GO analysis suggests that the biological function of CD56brightCD16- NK cells is mainly concentrated in positive regulation of chemotaxis, mononuclear cell migration and lymphocyte migration, in which the genes that play a major role are XCL1, XCL2, GPR183, CX3CR1, CD99, CCL5 and CCL4(Figure S3G-H).
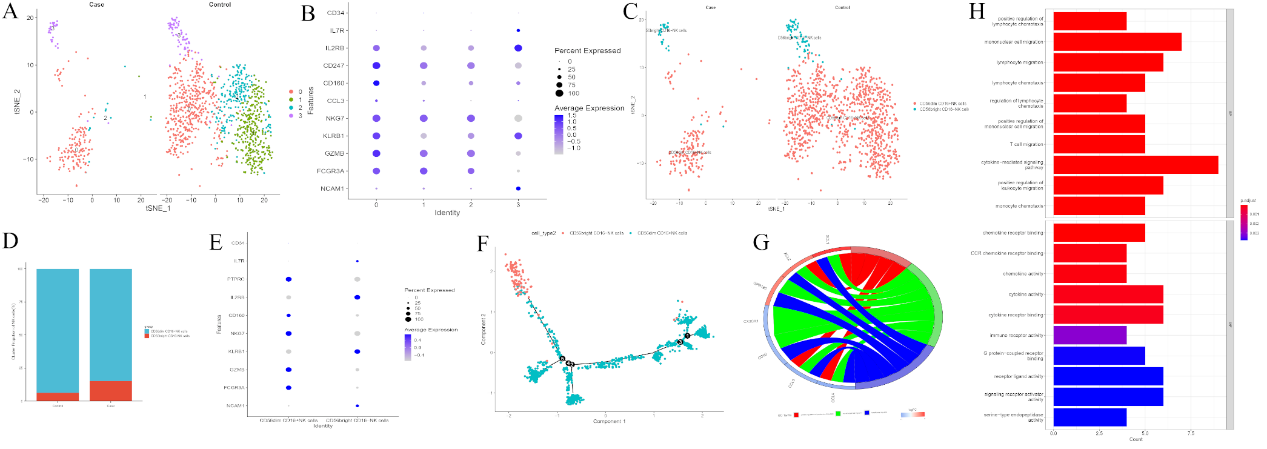


**Figure S3. Further cluster annotation of Natural killer cells**
